# Metal phthalocyanine: fullerene composite nanotubes via templating method for enhanced properties

**DOI:** 10.1186/s11671-015-0741-6

**Published:** 2015-02-06

**Authors:** Abdullah Haaziq Ahmad Makinudin, Muhamad Saipul Fakir, Azzuliani Supangat

**Affiliations:** Department of Physics, Low Dimensional Materials Research Centre, University of Malaya, Kuala Lumpur, 50603 Malaysia

**Keywords:** VOPcPhO, PC_71_BM, Composite nanotubes, Templating method

## Abstract

The use of templating method to synthesize the vanadyl 2,9,16,23-tetraphenoxy-29H,31H-phthalocyanine (VOPcPhO):[6,6]-phenyl C71 butyric acid methyl ester (PC_71_BM) composite nanotubes is presented here. VOPcPhO is a p-type material and PC_71_BM is an n-type material which acts as an electron donor and electron acceptor, respectively. Both materials have been studied due to their potential applications as solar energy converter and organic electronics. High-resolution transmission electron microscope (HRTEM) and field emission scanning electron microscope (FESEM) images have shown the replication of the porous template diameter of approximately 200 nm with a superior incorporation of both VOPcPhO and PC_71_BM. VOPcPhO:PC_71_BM composite nanotubes showed the significant properties improvement if compared over their bulk heterojunction counterpart. UV-vis spectra of composite nanotubes show a shift to a longer wavelength at the absorption peaks. Significant quenching has been attained by the photoluminescence spectra of VOPcPhO:PC_71_BM composite nanotubes which supports the redshift of UV-vis absorption spectra. Presumably, the photo-induced charge transfer and charge carrier dissociation can be enhanced from the VOPcPhO:PC_71_BM composite nanotubes rather than the bulk heterojunction.

## Background

Metal phthalocyanines have been widely studied for electronic device applications due to their notable chemical properties of high solubility in a variety of organic solvents and physical properties of efficient absorption of light in the visible region [1,2]. Metal phthalocyanine nanostructures such as nanotubes, nanoflowers, nanorods, nanowires, and nanoribbons [3-6] have shown the remarkable approaches and the emergence of versatile fabrication. A spin coating technique has been implemented in fabricating the electronic devices (such as sensors and solar cells) with the incorporation of metal phthalocyanine as bulk heterojunction structure [1,7-9]. Bulk heterojunction could be realized by blending the p- and n-type materials which then resulted to the interpenetrating structure. Understanding the process of morphological changes, light absorption, and charge carriers transfer between the merged p- and n-type materials continues to be the crucial studies. Of particular interest are the architecture and the creation of interfaces between the p- and n-type materials as it is reflected to the photo-induced charge carriers. Among of numerous techniques used in fabricating the novel architecture of nanostructured materials, uncomplicated technique of template-assisted method is of considerable interest [3-5,10-12]. In contrast to the interpenetrating bulk heterojunction structure synthesized by blending the p- and n-type materials, template-assisted method of porous alumina could produce a versatile and unique heterostructured nanostructure such as composite nanorods and nanotubes by a simple layer-by-layer approach [4,13]. Via a simple layer-by-layer approach, two dissimilar materials are incorporated together at a different time.

Recently, interest in fabricating the composite nanostructures that involves one of the metal phthalocyanines families, namely vanadyl 2,9,16,23-tetraphenoxy-29H,31H-phthalocyanine (VOPcPhO), has gained much attention. VOPcPhO possesses extraordinary features which can enable it to act as an electron donor (p-type) or an electron acceptor (n-type) when incorporated with other materials [1,3,4,9,14]. VOPcPhO appears as green to dark blue-green in color and is highly soluble in various organic solvents [9]. It is considered as a macro-cyclic compound where its structure consists of four isoindole units surrounding the center metal atom. VOPcPhO also exhibits wide absorption in UV-vis spectral region between 300 and 750 nm [1-4,7-9]. In addition, metal phthalocyanine is capable to act as a sensitizer of photo-induced electron transfer to acceptors, which can be efficiently achieved by incorporating it with the fullerene groups such as [6,6]-phenyl C61 butyric acid methyl ester (PC_61_BM) and [6,6]-phenyl C71 butyric acid methyl ester (PC_71_BM) [15]. The fullerene groups are considered to be the ideal electron acceptor materials in many organic-based devices. Fullerenes practically have an energetic deep-lying LUMO which endows the molecule with a high electron affinity relative to the numerous potential organic donors [15,16].

Although bulk heterojunction and composite nanostructures exhibited similar properties in light absorption, composite nanostructures (such as composite nanorods) have shown to have exceptional features on their optical properties if better light absorption is realized. This is due to the enhanced exertion on the large surface area produced by composite nanorods [4,5]. In this study, VOPcPhO and PC_71_BM are used as the electron donor (p-type) and electron acceptor (n-type), respectively. The C70-based fullerene is chosen over the C60 allotrope due to its photo absorption enhancement in a large energy scale [17]. The VOPcPhO:PC_71_BM composite nanotubes are fabricated via a templating method and are further characterized for their morphological, structural, and optical properties. Comparison between the composite nanotubes and bulk heterojunction is elaborated in consideration of their properties.

## Methods

Vanadyl 2,9,16,23-tetraphenoxy-29H,31H-phthalocyanine (VOPcPhO) (98% pure) and [6,6]-phenyl C71 butyric acid methyl ester (PC_71_BM) were purchased from Sigma-Aldrich (St. Louis, USA) and used without any alteration. Concentrations of 5 mg/ml of VOPcPhO and 5 mg/ml of PCBM solution were prepared separately in chloroform for a templating method of layer-by-layer assembly. A blend mixture of VOPcPhO and PC_71_BM with a ratio of 1:1 was also prepared with similar concentration of 5 mg/ml for the bulk heterojunction samples. Porous alumina templates (Whatman Anodisc, Sigma-Aldrich, St. Louis, USA) and glass substrates were used in synthesizing the composite nanotubes and bulk heterojunction, respectively. The glass substrates (Sail Brand, China) with dimensions of 1 × 1 cm and porous alumina templates with nominal pore diameter of 200 nm and thickness of 60 μm were both cleaned via sonication of acetone, ethanol, and de-ionized water for 15 min prior to oven drying at 60°C for 30 min and nitrogen blowing.

The composite nanotubes were prepared using spin coating and template immersion techniques. The template was first immersed in the VOPcPhO solution for 24 h prior to the drop-casted and spin-coated PC_71_BM solution. After the spin coating, the sample was annealed at 150°C for 1 min before the dissolution process is taken place. Template dissolution was done by immersing the template in 4 M sodium hydroxide (NaOH) for 12h. Meanwhile, for the preparation of bulk heterojunction, the blended mixture of VOPcPhO and PC_71_ BM was drop-casted onto the glass substrate and spin-coated at 1,000 rpm for 30 s before thermally annealed at 150°C for 1 min on a hot plate. Characterizations were performed via UV-vis spectroscope (Lambda 750, Perkin Elmer, Waltham, USA), photoluminescence spectroscope (Renishaw, Gloucestershire, UK), Raman spectroscope, field emission scanning electron microscope - energy dispersive X-ray spectroscope (FESEM-EDX) (JSM 7600-F, JEOL Ltd., Tokyo, Japan), and high resolution transmission electron microscope (HRTEM) (Hitachi HT7700, Japan).

## Results and discussion

### Formation of VOPcPhO:PC_71_BM composite nanotubes

**Figure 1:**
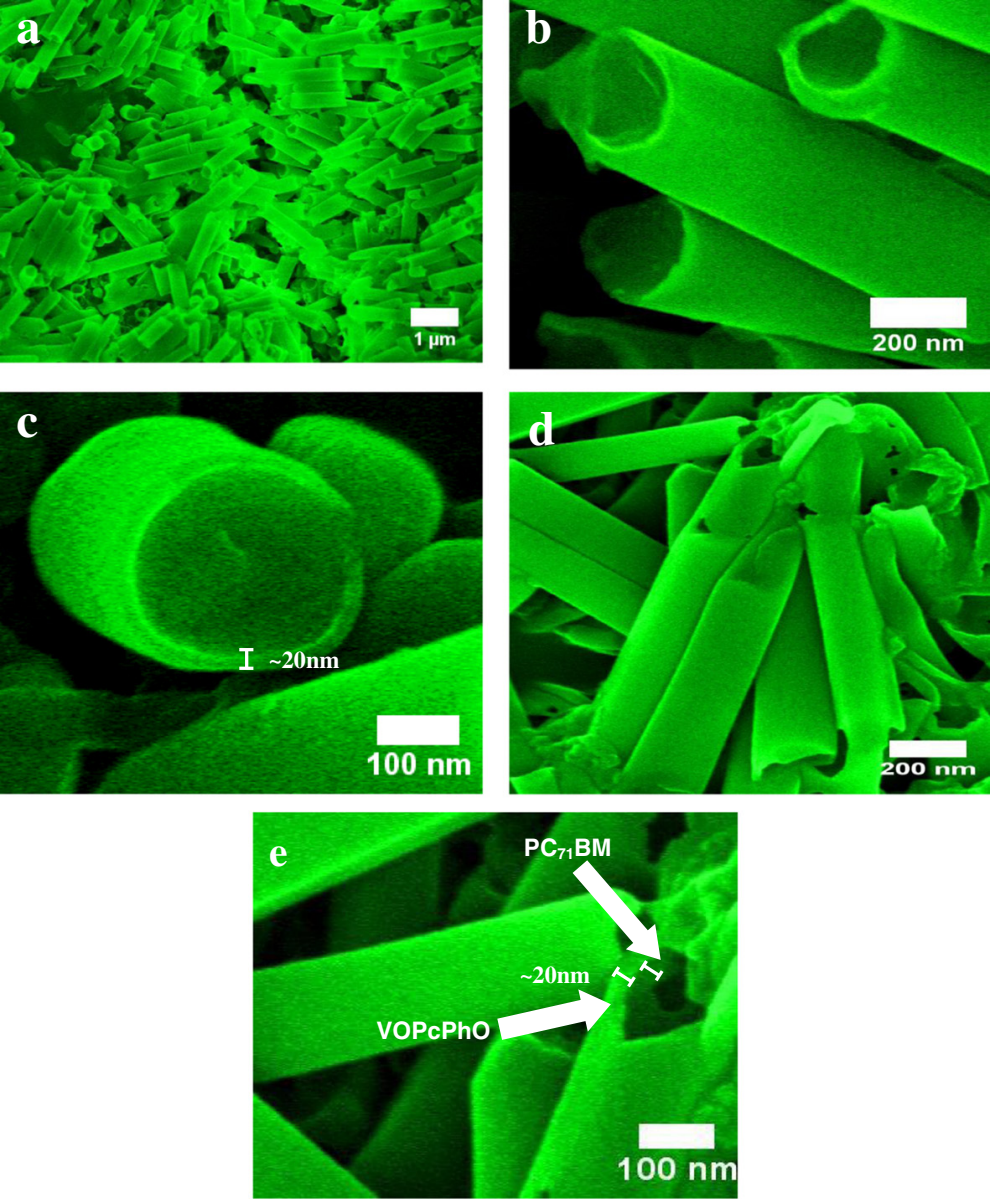
**FESEM images of VOPcPhO nanotubes (a-c) and VOPcPhO:PC**_**71**_ **BM composite nanotubes (d,e).**

FESEM images in Figure 1a-c shows the replication of the template's circular pores by the VOPcPhO nanotubes after 24 h of immersion. The diameter of the outer wall and wall thickness of VOPcPhO nanotubes is approximately 200 and 20 nm, respectively. This complete replication of the original alumina template suggested that the VOPcPhO solution has managed to fully infiltrate into the channel of porous template and creating a cylindrical coating within the inner wall of the channel. VOPcPhO solution is able to achieve the low viscosity properties due to its low solution concentration which further facilitates the infiltration of PC_71_BM solution. Figure 1d shows the FESEM images of VOPcPhO:PC_71_BM composite nanotubes which result from the infiltration of PC_71_BM into the VOPcPhO nanotubes. PC_71_BM has generated additional cylindrical layer inside the VOPcPhO nanotubes which led to the formation of composite nanotubes. As shown by the magnified image of VOPcPhO:PC_71_BM composite nanotubes in Figure 1e, two different regions with hole in the middle are erected. The brighter region (outer wall) is corresponded to the VOPcPhO layer, while the darker region is initiated by the PC_71_BM. Empty hollow region can be clearly seen lying in the middle of the composite nanotubes. The formation of composite nanotubes suggests that more interfaces can be created between the two dissimilar materials. Expectations on the further enhanced photo-induced charge transfer phenomena are rather high via the fabrication of composite nanotubes if compared with the bulk heterojunction.

**Figure 2:**
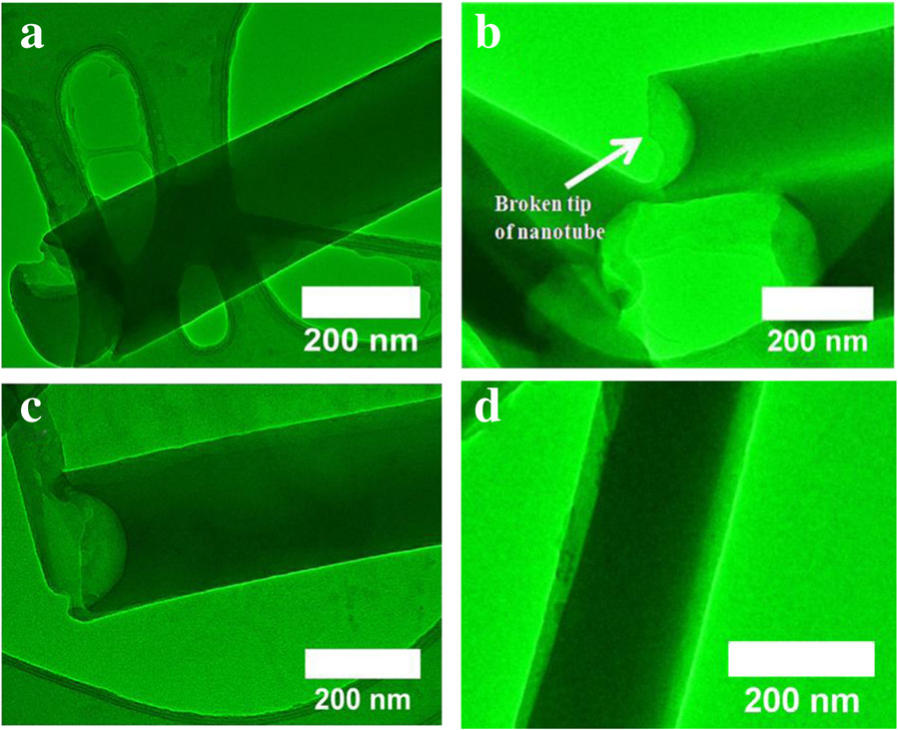
**HRTEM images of VOPcPhO nanotubes (a,b) and VOPcPhO:PC**_**71**_ **BM composite nanotubes (c,d).**

**Figure 3:**
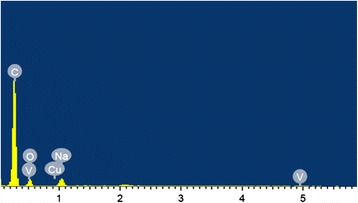
**EDX spectrum of VOPcPhO:PC**_**71**_ **BM composite nanotube.**

HRTEM images of the individual VOPcPhO nanotubes are shown in Figure 2a,b. From the images, it is clearly illustrated that the VOPcPhO nanotubes are constructed prior to the infiltration of PC_71_BM. These HRTEM images are correlated with those obtained in FESEM images with the identical outer diameters observed. The broken tube at the tip of VOPcPhO nanotubes supported the formation of nanotubes rather than nanorods. VOPcPhO solution has evidently created a thin cylindrical coating of approximately 20 nm over the porous channel. Twenty-four hours of immersion is sufficient for the formation of thin nanotubes' outer walls to initiate which then further allow the infiltration of other materials. The successful second infiltration is supported by TEM images shown in Figure 2c,d. Two dissimilar regions (light and dark) compose of VOPcPhO and PC_71_BM are clearly perceived from the TEM images of VOPcPhO:PC_71_BM composite nanotubes. VOPcPhO:PC_71_BM composite has successfully created a tubular shape nanotube via the immersion and spin coating technique of porous alumina templates. To further corroborate the existence of VOPcPhO:PC_71_BM composite, energy-dispersive X-ray spectroscope (EDX) analysis is performed and the spectrum is shown in Figure 3. The identified elements such as carbon (C), oxygen (O), and vanadium (V) support the presence of VOPcPhO:PC_71_BM composite, while other detected elements of sodium (Na) and copper (Cu) correspond to the sodium hydroxide (dissolution solvent) and sample holder, respectively.

**Figure 4:**
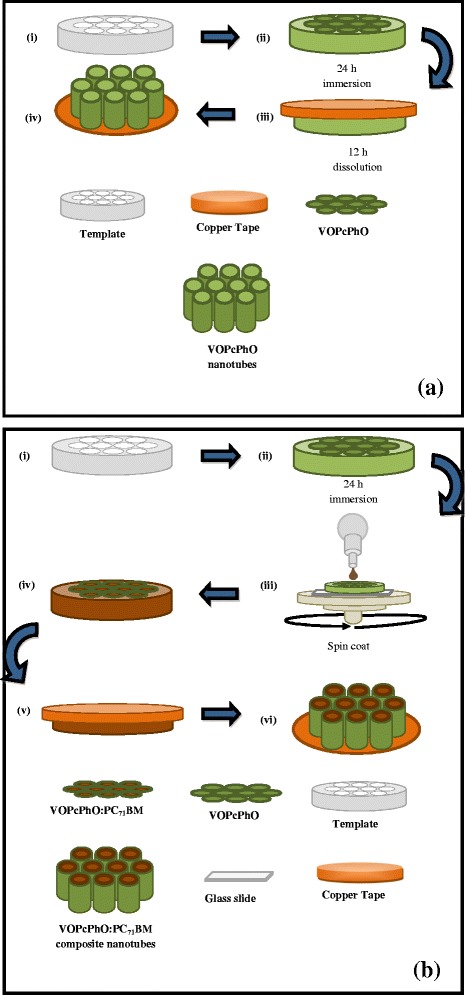
**Schematic illustrations on the formation of VOPcPhO nanotubes (a) and VOPcPhO:PC**_**71**_ **BM composite nanotubes (b).**

Schematic illustration of the proposed formation of VOPcPhO nanotubes and VOPcPhO:PC_71_BM composite nanotubes is depicted in Figure 4a,b, respectively. As shown in Figure 4a, the alumina template was first cleaned (i) prior to 24 h of immersion (ii). After 24 h of immersion, the sample was directly annealed at 150°C for 1 min. In order to ease the FESEM characterization process, sample was stuck upside down on copper tape due to the high conductive properties of the tape. The stuck sample was dissolved in NaOH for 12 h to remove the template (iii). Wetting and surface tension properties hold by VOPcPhO solution and template have steered to the formation of VOPcPhO nanotubes (iv). The immersion process has sanctioned the spreading of solution over the template's wall by creating an approximately 20 nm of wall thickness. The formation of VOPcPhO: PC_71_BM composite nanotubes are shown in Figure 4b. The first two steps (Figure 4b(i,ii)) were similar to that in Figure 4a(i,ii). Before further infiltration of PC_71_BM into the VOPcPhO nanotubes via spin coating technique (iii), the existing sample was thermally annealed. The main reason of using spin coating as a technique to infiltrate the PC_71_BM is due to the use of the chloroform as a solvent. Since the VOPcPhO is also soluble in chloroform, second immersion of VOPcPhO (template) into the PC_71_BM solution will only eradicate the initial nanotube wall created by VOPcPhO. Consequently, PC_71_BM nanotubes will be formed rather than the VOPcPhO: PC_71_BM composite nanotubes. In contrast, implementing the spin coating for the PC_71_BM infiltration could herald to the formation of composite nanotubes (iv). The sample will then be glued onto the copper tape prior to 12 h of template dissolution (v). The end product of VOPcPhO: PC_71_BM composite nanotubes is created (vi) due to the compatible wetting properties preserved between VOPcPhO nanotubes (surface) and PC_71_BM (solution) during the infiltration.

### Optical properties

**Figure 5:**
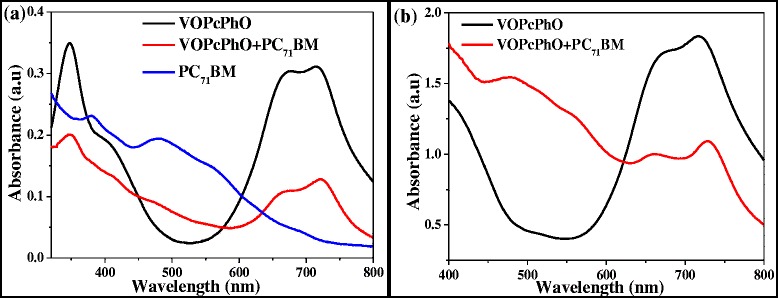
**UV-vis absorption spectra.** Comparison of UV-vis spectra of VOPcPhO thin film and VOPcPhO:PC_71_BM bulk heterojunction **(a)** and VOPcPhO nanotubes and VOPcPhO:PC_71_BM composite nanotubes **(b)**.

**Figure 6:**
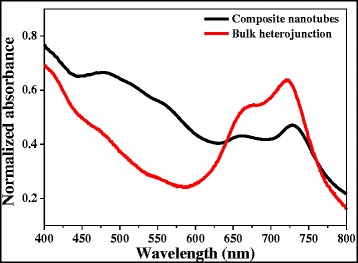
**UV-vis absorption spectra of VOPcPhO:PC**_**71**_ **BM composite nanotubes and bulk heterojunction.**

Figure 5a shows the absorption spectra of VOPcPhO thin film, PC_71_BM thin film, and VOPcPhO:PC_71_BM bulk heterojunction. As seen in UV-vis spectrum, the VOPcPhO has portrayed the significant peaks absorption between 300 and 750 nm. The peak absorption of VOPcPhO thin film is located at 348 nm which assigned as Soret band (B band) and at 677 and 713 nm of Q band [2-4,8,9,18]. UV-vis spectrum of VOPcPhO thin film portrayed an extensive valley between 400 and 600 nm which in the UV-vis spectrum of PC_71_BM thin film shows its main peak absorption positioned within this valley. It can be observed that by incorporating the two materials together, a broader absorption at the first peak of Q band can be realized. The main peaks of VOPcPhO:PC_71_BM bulk heterojunction spectrum lie at 346 nm (B band), 677 nm and 720 nm (Q band). Figure 5b shows the UV-vis absorption spectra of VOPcPhO nanotubes and VOPcPhO:PC_71_BM composite nanotubes. UV-vis absorption spectrum of VOPcPhO nanotubes shows significant improvement at the second peak of Q band. VOPcPhO nanotubes have attained higher absorption intensity if compared to their thin film. In addition, VOPcPhO:PC_71_BM composite nanotubes demonstrate a better incorporation between the two components. Absorption peak at 477 nm which is due to PC_71_BM can be clearly seen from the VOPcPhO:PC_71_BM composite nanotube spectrum. However, the peak assigned for PC_71_BM is not quite visible when the VOPcPhO:PC_71_BM is synthesized as bulk heterojunction. Comparison of UV-vis absorption spectrum between bulk heterojunction and composite nanotubes is shown in Figure 6. Composite nanotubes exhibit a redshift at their peaks in comparison to the bulk heterojunction. Existence of a slight change to the longer wavelength at the second peak of Q band is shown by the composite nanotubes. The second peak of Q band of VOPcPhO:PC_71_BM has shifted from 720 to 728 nm when their formation is altered from bulk heterojunction to composite nanotubes. This peak has experienced the absorption transition that occurred from the shorter to the longer wavelength by only tuning the architecture of materials. Postulation on the dependency between photon absorption and architecture of materials in improving its optical properties can be rather acceptable. The second π-π* transition on the phthalocyanine macro-cycle [3] is more dominant with the composite nanotubes formation which peak absorption transition has been enhanced from 713 to 728 nm. This transition has supported well to the incorporation of VOPcPhO and PC_71_BM that has been synthesized via a templating method. A templating method could provide a facile fabrication without the existence of intricate or affluent system. Additionally, wider peak absorption at the first peak of Q band is noticed in the UV-vis absorption spectrum of VOPcPhO:PC_71_BM composite nanotubes. This broader Q band could be due to the well distributed composite nanotubes shape between VOPcPhO and PC_71_BM. It can be evident from the spectrum that both the nanotubes and thin film composite exhibit a stronger peak at the second peak of the Q band. The equal activities of the first and second π-π* transition on the phthalocyanine macro-cycle, which can be seen from their equal peak absorption intensity, are achieved by VOPcPhO:PC_71_BM composite nanotubes.

**Figure 7:**
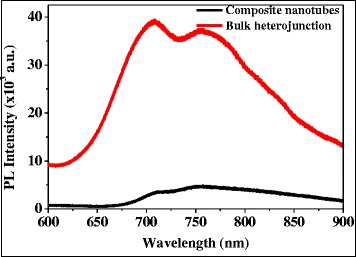
**Photoluminescence spectra of VOPcPhO:PC**_**71**_ **BM composite nanotubes and bulk heterojunction.**

The photoluminescence (PL) spectra of VOPcPhO:PC_71_BM bulk heterojunction and composite nanotubes are plotted in Figure 7. These PL spectra are obtained by an excitation wavelength of 325 nm which yield the range of wavelength between 600 and 900 nm. If compared to bulk heterojunction, PL spectrum suggests that the composite nanotube has exhibited a better photo-induced charge transfer between the donor/acceptor interfaces. This can be proven by the significant quenching phenomena shown in PL spectrum of composite nanotubes. As reported elsewhere, VOPcPhO has bipolar transport capabilities [9] which can act as either donor (p-type) or acceptor (n-type) material. Due to the compatible HOMO and LUMO values between VOPcPhO (3.32 and 5.33 eV) and PC_71_BM (3.9 and 6.0 eV), their structure as a donor/acceptor system can be accomplished. The efficient photon-induced charge transfer between VOPcPhO/PC_71_BM systems is one of the most significant characteristics of effective charge carriers' separation.

### Structural properties

**Figure 8:**
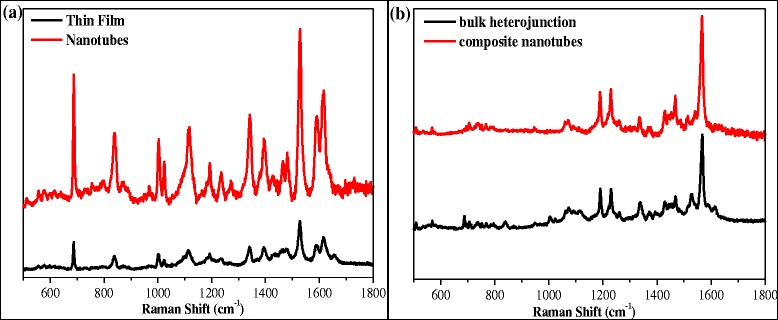
**Raman spectra.** Comparison of Raman spectra of VOPcPhO thin film and nanotubes **(a)** and VOPcPhO:PC_71_BM bulk heterojunction and composite nanotubes **(b)**.

Figure 8a,b shows the Raman spectra of VOPcPhO thin film versus nanotubes and Raman spectra of VOPcPhO:PC_71_BM bulk heterojunction versus composite nanotubes, respectively. These spectra have exhibited some differences in their intensities at the different peaks. The peaks that are presented in VOPcPhO thin film and nanotubes, however, are diminished with the formation of bulk heterojunction and composite nanotubes. The presence of PC_71_BM in the donor/acceptor system could be the main reason to which alteration of the molecular structure may have been occurred within the system. Assignments and changes in the wavenumber band between VOPcPhO thin film and nanotubes and VOPcPhO:PC_71_BM bulk heterojunction and composite nanotubes are tabulated in Table 1. Similar peaks between VOPcPhO thin film and nanotubes can be observed at 686; 838; 1,002; 1,023; 1,192; 1,340; 1,393; 1,527; 1,566; 1,590; and 1,616 cm^−1^ which are assigned for macrocycle bending and stretching, benzene ring breathing, C-H bending, pyrrole stretching, ring stretching, and C = C stretching, with slightly upward shift approximately 4 cm^−1^ that can be observed from the VOPcPhO nanotubes of the certain peaks at 1,117; 1,236; 1,464; and 1,481 cm^−1^. Peaks at 838; 1,004; 1,025; and 1,614 cm^−1^ which represent the macro-cycle stretching, benzene ring breathing, C-H bending, and C = C stretching, respectively, are missing in the VOPcPhO:PC_71_BM composite nanotubes as compared to the bulk heterojunction. This could be due to the existence of the significant variation in the ordering structure of composite nanotubes. The formation of composite nanotubes is expected to alter the molecular structure of VOPcPhO and PC_71_BM. Disappearance of peaks at 1,113/1,117 and 1,477/1,481 cm^−1^ occurred after the PC_71_BM was added to the VOPcPhO:PC_71_BM system. These two peaks which are assigned for C-H bending and ring stretching are recorded in VOPcPhO nanotubes and VOPcPhO thin film but neither in VOPcPhO:PC_71_BM bulk heterojunction or VOPcPhO:PC_71_BM composite nanotubes. The peaks' disappearance due to the incorporation of PC_71_BM shows that the effect of VOPcPhO on the Raman modes of VOPcPhO:PC_71_BM is too small to be considered influential [9].

**Table 1:** **Raman peak position of VOPcPhO and VOPcPhO:PC**_**71**_ **BM**

**Raman shift (cm** ^**−1**^ **)**				
**VOPcPhO**		**VOPcPhO:PC**_**71**_ **BM**		**Assignments**
**Thin film**	**Nanotubes**	**Bulk heterojunction**	**Composite nanotubes**	
686	687	687	703	Macrocycle breathing
838	836	838	-	Macrocycle stretching
-	-	948	946	Ring breathing
1,002	1,003	1,004	-	Benzene ring breathing
1,023	1,024	1,025	-	C-H bending
-	-	1,061	1,059	Ring vibration
-	-	1,073	1,071	Ring stretch
1,113	1,117	-	-	C-H bending
1,192	1,193	1,189	1,189	C-H bending
1,232	1,236	1,230	1,230	C-H bending
1,340	1,341	1,338	1,335	Pyrrole stretching
1,393	1,393	1,392	1,392	Ring stretching
-	-	1,444	1,444	Ring stretching
1,460	1,464	1,468	1,468	Ring stretching
1,477	1,481	-	-	Ring stretching
1,527	1,528	1,527	1,515	Pyrrole stretching
1,566	1,566	1,568	1,567	C = C stretching
1,590	1,591	1,592	1,592	C = C stretching
1,616	1,616	1,614	-	C = C stretching

## Conclusions

In this work, VOPcPhO:PC_71_BM composite nanotubes have been synthesized via a templating method of immersion and spin coating. VOPcPhO:PC_71_BM composite nanotubes have added advantages in terms of their morphological, structural, and optical properties if compared with the bulk heterojunction. Charge carrier separation at the p-n interfaces is augmented in a composite nanotube rather than in bulk heterojunction.

## Abbreviations

EDX: energy-dispersive X-ray spectroscope
FESEM: field emission scanning electron microscopy
HOMO: highest occupied molecular orbital
HRTEM: high resolution transmission electron microscopy
LUMO: lowest unoccupied molecular orbital
NaOH: sodium hydroxide
PC_71_BM: [6,6]-phenyl C_71_-butyric acid methyl ester
PL: photoluminescence
VOPcPhO: vanadyl 2,9,16,23-tetraphenoxy-29H,31H-phthalocyanine
